# Post-Machining Deformations of Thin-Walled Elements Made of EN AW-2024 T351 Aluminum Alloy as Regards the Mechanical Properties of the Applied, Rolled Semi-Finished Products

**DOI:** 10.3390/ma14247591

**Published:** 2021-12-10

**Authors:** Magdalena Zawada-Michałowska, Paweł Pieśko

**Affiliations:** Faculty of Mechanical Engineering, Lublin University of Technology, 20-618 Lublin, Poland; p.piesko@pollub.pl

**Keywords:** deformation, thin-walled elements, milling, mechanical properties, aluminum alloy, semi-finished product

## Abstract

The paper presents an evaluation of post-machining deformations of thin-walled elements as regards the mechanical properties of the applied, rolled semi-finished products. Nowadays, wrought aluminum alloys, supplied primarily in the form of rolled plates, are widely applied in the production of thin-walled integral parts. Considering the high requirements for materials, especially in the aviation sector, it is important to be aware of their mechanical properties and for semi-finished products delivered after plastic working to take into account the so-called “technological history” concerning, inter alia, the direction of rolling. The study focused on determining the influence of the ratio of the tension direction to the rolling direction on the selected mechanical properties of the EN AW-2024 T351 aluminum alloy depending on the sample thickness and its relation to the deformation of thin-walled parts. Based on the obtained results, it was found that the sample thickness and the ratio of the tension direction to the rolling direction affected the mechanical properties of the selected aluminum alloy, which in turn translated into post-machining deformations. Summarizing, the textured surface layer had a significant impact on the mentioned deformation. Greater deformations were noted for samples made of a semi-finished product with a thickness of 5 mm in comparison to 12 mm. It was the result of the influence of the surface layer, which at lower thickness had a higher percentage of contents than in thicker samples.

## 1. Introduction

Aluminum ranks second (after iron) as the most commonly used metal, which proves its great technical importance [1]. Due to the low strength of pure aluminum, its alloys are commonly used and they are divided into wrought alloys and cast alloys. However, wrought alloys are applied with more frequency and the range of their application is constantly increasing [2,3,4,5,6].

The advantages of aluminum alloys include, in particular, the following [7,8]:

- relatively low density (they are included in the group of light materials);
- high strength-to-weight ratio; and
- resistance to corrosion.

The large variety of aluminum alloys and the differences in their properties mean that they are used in many industries such as aviation [9,10,11] and automotive [12]. It is critical that the requirements for materials, especially in aviation, are very high; therefore, the exact knowledge of their properties, especially mechanical, is very important [13,14]. Material characteristics of various materials, including aluminum alloys, appear in a number of publications. They are mainly determined in the tensile test with many factors taken into account. Moreover, the correct design of elements requires understanding the mechanical parameters and the mechanism of deformation of the material subject to testing [15]. It is especially important in the case of wrought aluminum alloys, since rolled plates are used very often as semi-finished products for the production of parts, e.g., in aviation, and they have different properties depending on the direction. It is due to the rolling direction, anisotropy phenomenon, and residual stresses generated at the stage of the production of the semi-finished product [16,17,18,19].

The paper [15] presented a tensile test of the 7017 aluminum alloy depending on the strain rate and temperature as well as the implementation of the obtained data into the Johnson–Cook constitutive model. The authors of the paper [20] subjected the AlCu4Mg1 (EN AW-2024) duralumin to tensile tests, emphasizing the importance of the tensile test while selecting the material for engineering applications. Cadoni et al. [21] presented the experimental tensile tests of the AA7081 aluminum alloy (indication according to the Aluminium Association), where they analyzed the sensitivity of mechanical properties to the strain rate during tension and then used the obtained results to develop a constitutive Johnson–Cook model. The paper [22] discussed the relationship between the ultimate tensile strength *UTS R_m_* and the deformation rate for the aluminum alloy AA2139-T351 (indication also by the Aluminium Association). Additionally, a difference in mechanical properties was found depending on the tension direction in relation to the rolling direction. The ultimate tensile strength *UTS R_m_* was received while tension in the rolling direction fluctuated in the range of 497–502 MPa, whereas, for the transverse direction, the ultimate tensile strength *UTS R_m_* was 473–480 MPa. Chen et al. [23] tested four aluminum alloys, AA6060, AA6082, AA7003, and AA7108 (Aluminium Association designation) in the T6 state, used in the automotive industry. A wide range of strain rates and the relationship between stress, strain, and anisotropy of properties were investigated. It was found that the AA6082-T6, AA7003-T6, and AA7108-T6 alloys are characterized by a clear anisotropy of strength with the lowest value in the direction of 45° in relation to the extrusion direction. The strength in the direction of 90° was slightly lower than in the extrusion direction. On the other hand, the anisotropy of strength for the AA6060-T6 alloy was minor. The paper [24] presented the analysis of deformation under the influence of tensile stresses for widely used alloys, i.e.,: AW-5083 and AW-2024. The author of the doctoral dissertation [25] showed the stress-elongation characteristics obtained for the EN AC-43000 cast aluminum alloy and the EN AW-6082 wrought aluminum alloy. Analyzing the machinability of both materials, the EN AC-43000 alloy was classified in group 2, while the EN AW-6082 alloy, due to its high plasticity, was in group 1. Based on the results, it was found that the tested materials differ in their mechanical properties. The EN AC-43000 cast alloy is characterized by a higher Young’s modulus *E* = 74.5 MPa, but with lower ultimate tensile strength *UTS R_m_* = 121.7 MPa than the EN AW-6082 wrought alloy, for which Young’s modulus is *E* = 65.7 MPa, and the ultimate tensile strength *UTS* amounts to *R_m_* = 204.9 MPa (almost 70% more than for cast alloy). The values of individual mechanical indexes of the material may differ depending on the applied parameters of the tensile test [20,22].

Currently, aluminum alloys are widely used in the aviation and automotive industries. This is related to the tendency to minimize the mass of manufactured parts. The technological difficulties associated, among others, with machining of thin-walled elements are very important issues both from a scientific and practical point of view [11,26]. One of the main problems is the formation of undesirable deformations of thin-walled elements after machining and removal of the forces clamping the workpiece in the clamping device [27]. Straitening the number of components of individual assemblies determines the ever wider use of the so-called integral (structural) thin-walled elements characterized by a uniform structure and low weight compared to the usually large overall dimensions. Currently, there is also a tendency to simplify semi-finished products; hence, such structures are mainly made of monolithic plates rolled from light metal alloys, such as aluminum. In addition, a very large amount of resulting chips, even more than 95% of the semi-finished product mass, implies the use of high-performance milling, i.e.,: High-Performance Cutting and High-Speed Cutting, which are a part of the trend entering in reducing production costs by removing the allowances faster than in traditional way [9,28,29,30,31].

The formation of post-machining deformations of thin-walled elements is a complex process, dependent on many factors. One of the main reasons is the residual stresses generated at each stage of the technological process (semi-finished product production, machining, heat treatment). The most common models of residual stresses after machining are thermal and mechanical models. The first one is dedicated for abrasive machining and High-Speed Cutting, while the second one is typical for machining [17,18,32]. Deformations of thin-walled elements are also difficult to predict because, apart from residual stresses, they result from a number of other factors such as temperature, clamping force, cutting force, etc. [17,33,34,35,36]. In industry, to reduce residual stresses, for example, heat treatment, seasoning, and vibration methods are used. However, these are additional operations, significantly increasing costs and extending production time. In addition, their use is problematic for large-size parts. Therefore, the industry aims to eliminate such operations and it is looking for other methods to minimize the deformation of thin-walled elements occurring after milling [37,38,39].

For the production of thin-walled elements from monolithic rolled plates, it is also important to take into account the properties’ anisotropy of the materials used, which contributes to the abovementioned post-machining deformations. In addition, technological recommendations may differ depending on the alloy being machined. This is due to the different properties and machinability of individual materials. It should also be noted that the dynamic development of the automotive and aviation sectors influences the continuous progress in the development of new materials, including also light metal alloys, and in improving the technology of their production and formation by machining as well [16,40,41,42].

In conclusion, on the basis of the literature review, it was found that the mechanical properties of the material used may affect post-machining deformations of thin-walled elements. An important aspect is also the anisotropy of the mentioned properties and its influence on the deformation of such parts. This paper is a continuation of the publication cycle related to deformations of thin-walled elements. It discusses the impact of the material strength properties on the deformations of these parts. In previous authors’ papers (e.g., [11,19,38,41,43,44]), attention was concentrated on the possibility of deformation minimization by using appropriately selected solutions such as, for example, milling strategy, pre-machining, or technological parameters. In the present paper, the authors were focused on the cause-and-effect relationship analysis and they made an attempt to relate the resulting deformations with the mechanical properties of materials.

The aim of the study was to evaluate deformations of post-machining thin-walled elements in terms of the mechanical properties of the rolled semi-finished products used. The practical aim was also to develop, on the basis of scientific research, technological guidelines that can find technical application and minimize the resulting deformations.

## 2. Materials and Methods

### 2.1. Tensile Tests

Based on the analysis of the current state of knowledge concerning the deformation of thin-walled elements after milling and issues related to the planning of the experiment, the methodology of experimental research was developed.

The own research was divided into two parts. The first part referred to the static tensile test aimed at determining the material characteristics of the EN AW-2024 aluminum alloy in the T351 condition, for various thicknesses of rolled sheets and plates, taking into account their rolling direction. The second part of the research involved the examination of the resulting post-machining deformations of thin-walled test samples.

**Figure 1:**
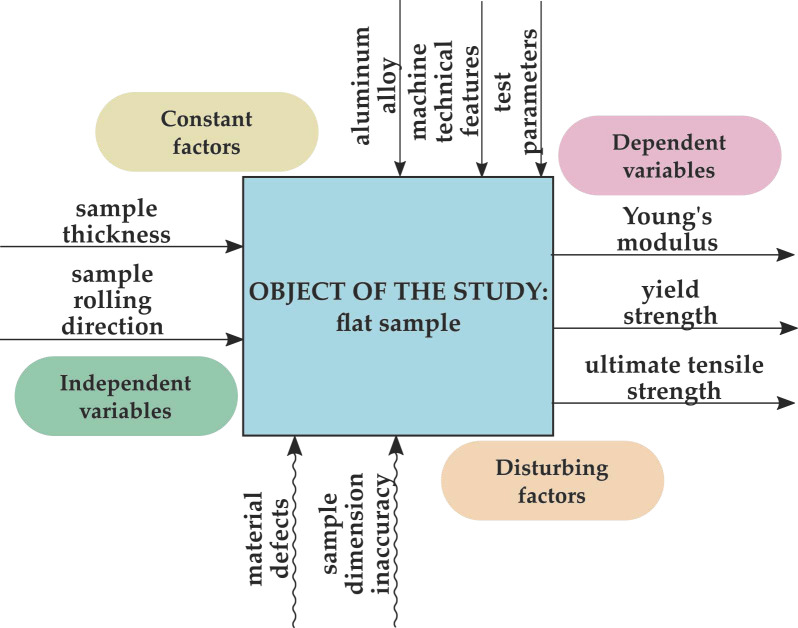
Model research object, first part.

During the static tensile test, flat samples were the object of the study. The model of the tensile test object along with the factors influencing it and the analyzed variables is presented in Figure 1.

The independent variables included the thickness and the direction of rolling of the samples. The analyzed dependent factors were Young’s modulus *E*, yield strength *R_p_*_0.2_, and ultimate tensile strength *UTS R_m_*. The constant factors affecting the object were EN AW-2024 T351 aluminum alloy, technical features of the machine, and test parameters, while the disturbances included dimensional inaccuracy of samples and material defects.

In order to determine the significance of the influence of thickness and rolling direction of samples on Young’s modulus *E* and ultimate tensile strength *UTS R_m_*, a complete, randomized, statistical program was used [45].

The experiment was carried out for the following five different thicknesses of samples:

- 1 mm;
- 1.2 mm;
- 1.6 mm;
- 4.85 mm; and
- 10.2 mm.

The selection of the sample thicknesses for both tensile tests and deformation research resulted from many years of project work performed at the Lublin University of Technology in cooperation with aviation companies.

The directions of rolling were also analyzed, i.e.:

- tension direction perpendicular to rolling direction (perpendicular direction); and
- tension direction parallel to rolling direction (parallel direction).

The samples were made of EN AW-2024 T351 multi-component wrought aluminum alloy. The material was characterized by relatively high strength and good machinability, but also low corrosion resistance and limited weldability. Taking into account that the problem of thin-walled element deformations is reported mainly by aviation companies, it was decided to choose a material that is widely used in aviation. T351 condition means that the material was heat-treated, stress-relieved by controlled tensile stress, and then naturally aged. The chemical composition and selected properties of the EN AW-2024 T351 alloy are presented in Table 1.

In order to determine the material characteristics of the tested EN AW-2024 T351 aluminum alloy, a static tensile test was carried out in accordance with PN-EN ISO 6892-1:2016-09 standard [48]. The Zwick/Roell Z150 testing machine (ZwickRoell GmbH & Co. KG, Ulm, Germany) was used in the research.

The aim of the tensile tests was to determine the effect of rolling and the ratio of the tension direction to the rolling direction on the mechanical properties of the EN AW-2024 T351 aluminum alloy, depending on the thickness of the element. There was a suspicion that the textured surface layer had a significant influence on the Young’s modulus *E* and ultimate tensile strength *UTS R_m_* for relatively thin samples. The presented assumption resulted from the conducted preliminary tests, during which a change in the mechanical properties of the samples depending on their thickness and anisotropy was found.

The basic parameters of the Zwick/Roell Z150 testing machine [49] were:

- maximum breaking force: 150 kN;
- traverse speed range: 0.0005–900 mm/min;
- temperature range: from −40 to +240 °C (with a thermal chamber);
- drive resolution: 0.0123 μm; and
- extensometer compliant with PN-EN ISO 9513:2013-06 standard [50].

The determination of the sample size was carried out on the basis of the following procedure. First, it was checked with a test *W* Shapiro–Wilk, normality of the distribution of the analyzed variables. Sample size *n* was determined on the basis of the conducted preliminary research and dependence (1) [45]:

(1) $$n=\frac{t_{\left(\alpha ;f\right)}^{2}S_{x}^{2}}{A^{2}},$$

where $S_{x}^{2}$ is the variance of the examined feature determined on the basis of the results of preliminary tests, *A* is the accuracy of assessment, *t* is the critical value of t-Student’s distribution, α is the significance level, and *f* is the number of degrees of freedom.

Initial tensile tests were performed for samples with a thickness of 10.2 mm, where the tension direction was parallel to the rolling direction. The static tensile test was repeated eight times. The statistical dispersion of obtained results for the Young’s modulus *E* and ultimate tensile strength *UTS R_m_* was analyzed and determined. The accuracy of assessment *A* assumed at the level of 5% of the received mean value. Based on the adopted significance level *α* = 0.05 and the number of degrees of freedom equal to *f* = 7, the coefficient was read from the t-Student and was equal *t* = 2.365. Table 2 shows the calculation of the size of sample *n* for further tensile tests.

In the proper tensile tests, the sample size *n* = 10 was adopted for all analyzed variables.

### 2.2. Deformation Research

**Figure 2:**
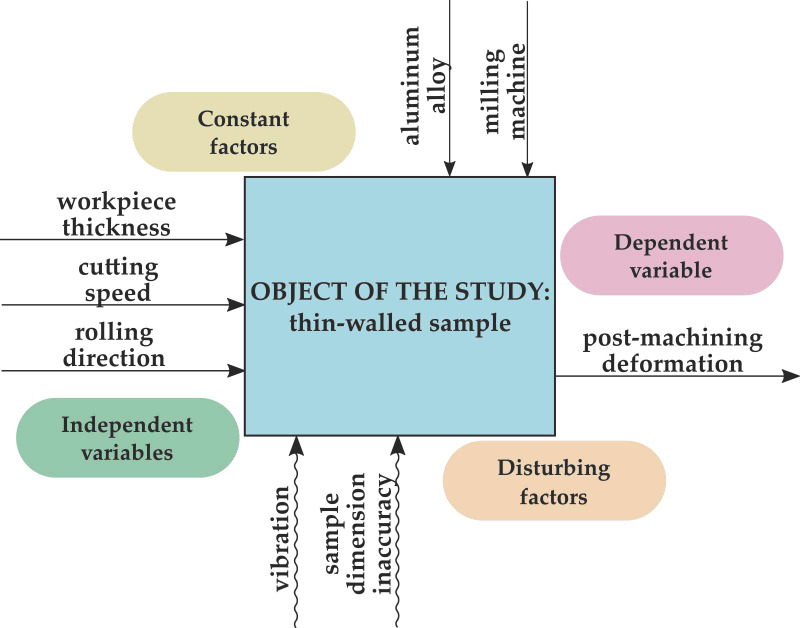
Model of the research object, second part.

In the second part, thin-walled samples were the object of the study. The model of the research object is presented in Figure 2.

The independent variables were cutting speed (corresponding to conventional and high-speed cutting), sample thickness, and rolling direction, while the dependent variable was post-machining deformation. Milling machine and aluminum alloy were defined as constant factors. The disturbing factors included vibrations and dimensional inaccuracy of the samples. In order to determine the significance of the influence of the studied independent variables on the dependent variable, a complete, randomized, statistical program was also used [45].

The tests were carried out for the following thicknesses of the semi-finished product:

- 5 mm; and
- 12 mm.

In addition, cutting speeds *v_c_* were used for finishing, such as:

- *v_c_* = 200 m/min (corresponding to conventional processing); and
- *v_c_* = 1200 m/min (corresponding to High-Speed Cutting).

The independent variable was also the rolling direction, namely, the ratio of the cutting tool feed direction to the rolling direction:

- tool feed direction perpendicular to rolling direction; and
- tool feed direction parallel to rolling direction.

**Figure 3:**
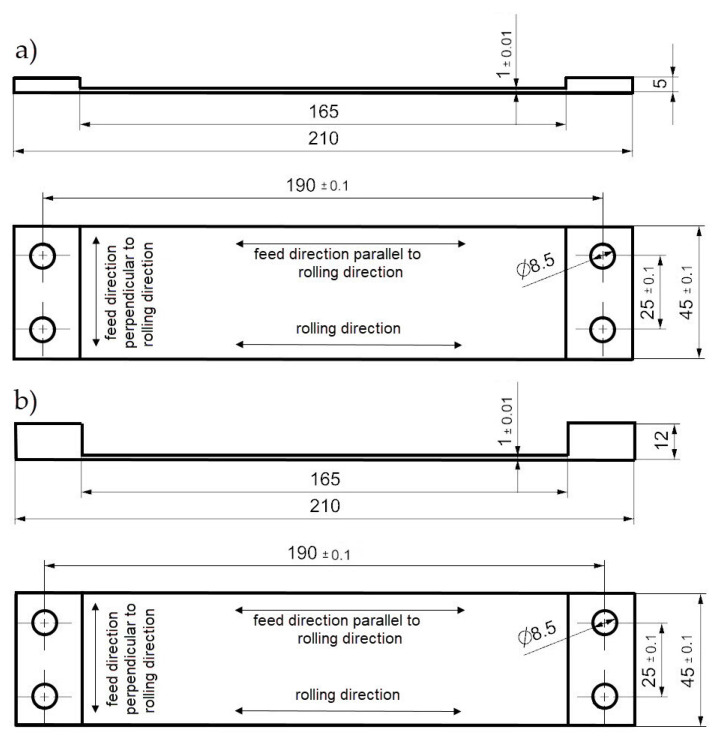
View of the sample after milling: (**a**) semi-finished product thickness: 5 mm, (**b**) semi-finished product thickness: 12 mm.

In the tests, the semi-finished products were the rolled plates with thicknesses of 5 mm and 12 mm made of the EN AW-2024 T351 alloy. A WaterJet Combo machine tool by ECKERT (Eckert AS Sp. Z o.o., Legnica, Poland) was used to cut the cuboidal samples. Figure 3 shows the view of the samples after machining. In both cases, the relief had a length of 165 mm and a width of 45 mm, as well as the thickness of the bottom of 1 mm. The relationships between the cutting tool feed direction and the rolling direction were also determined.

The tests were carried out on the Avia VMC 800 HS vertical machining center (Fabryka Obrabiarek Precyzyjnych AVIA S.A., Warsaw, Poland). The following end mills were used in the research:

- Kennametal (Kennametal, Pittsburgh, PA, USA) indexable milling cutter with cutting inserts, HPC, and
- Sandvik (Sandvik, Stockholm, Sweden) monolithic milling cutter, finishing (*v_c_* = 200 m/min and *v_c_* = 1200 m/min).

The technical specifications of the tools used are presented in Table 3.

Taking into account the grip part of the tools and the machine spindle taper, the appropriate Haimer (Haimer GmbH, Igenhausen, Germany) HSK 63A holders were selected. For the Kennametal milling cutter, the Weldon-type holder (A63.000.25) was used, while for the Sandvik milling cutter, a heat-shrinkable holder (A63.144.16) was used. They were balanced in accordance with the G 2.5 class at 25,000 rpm, as per ISO 1940-1:2003 [53].

Rough machining was carried out in all cases using the High-Performance Cutting and technological parameters such as *v_c_* = 1000 m/min, *a_p_* = 3.5 mm, and *f_z_* = 0.1 mm/tooth. The number of passes for a plate with a thickness of 5 mm was one, while for a plate with a thickness of 12 mm, the numbers were three. The cutting parameters for finishing are listed in Table 4. In this case, the number of tool passes was one. The cutting speed *v_c_* was the independent variable, which corresponded to conventional and High-Speed Cutting, respectively. Roughing and finishing were wet machined using a MobilCut 230 (ExxonMobil Poland sp. z o.o., Warsaw, Poland) coolant.

In order to ensure the correct mounting and fixation of the object during milling, it was necessary to design and manufacture a special holder made of EN AW-7075 T6 aluminum alloy.

For the measurement of absolute deformations, a differential method was used with a diatest Sylvac CL44 amplifying sensor (Sylvac S.A., Yverdon-les-Bains, Switzerland), enabling measurement in two directions (+, −). The instrument has a horizontal plunger and has a low pressure of 0.07 N, which makes it suitable for measuring thin walls.

The measuring station consisted of a Sylvac CL44 sensor mounted in a magnetic holder, which was then placed on the Avia VMC 800 HS headstock. The connection to the computer was made via an optoelectronic link. Syl Connect V1.072 software was used to save and analyze the results of the measurement.

Measurements were performed both before and after unfastening the sample from the clamping device. However, the paper presents only the results after unfastening. The measurement was carried out in the middle plane spaced by 22.5 mm from the edge of the sample. In the measuring section *l* = 165 mm, a total of 67 points were recorded. The sensor was reset against the vertical wall at *l* = 0 mm.

The basic technical parameters of the Sylvac CL44 digital sensor are summarized in Table 5.

Determining the sample size *n* for deformation testing using the differential method, the procedure was analogous to that for tensile tests. Initial tests were carried out for two directions of rolling and conventional finishing and semi-finished product thickness of 5 mm. The obtained results were analyzed by calculating standard deviation and variance. Accuracy of assessment *A* was assumed at the level of 5% of the mean value. Based on the adopted significance level *α* = 0.05 and the number of degrees of freedom equal to *f* = 4, the coefficient, from the t-Student, was *t* = 2.776. Determining the sample size *n* for the study of relative and absolute deformations is presented in Table 6.

With sample size *n* for the study of absolute deformations, it was assumed at the level of *n* = 5 for each analyzed configuration.

## 3. Results

### 3.1. Tensile Tests

**Figure 4:**
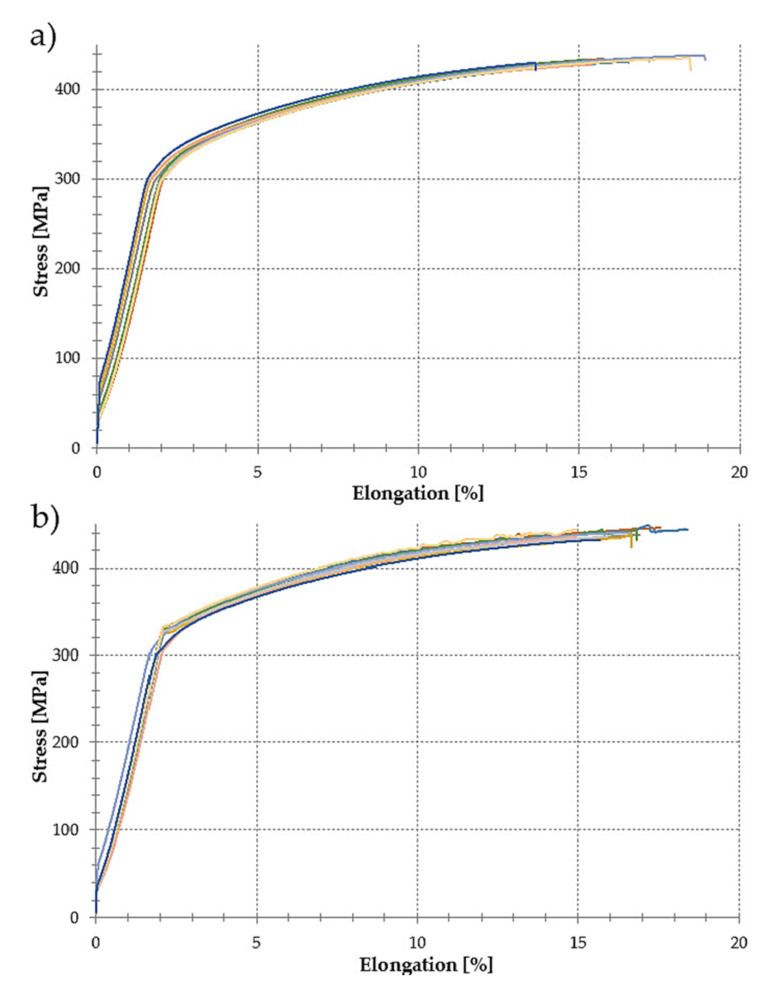
Material characteristics of the EN AW-2024 T351 alloy obtained for samples with a thickness of 1 mm and the ratio of the tension direction to the rolling direction: (**a**) perpendicular, (**b**) parallel.

The mechanical properties of the EN AW-2024 T351 aluminum alloy were determined on the basis of the results obtained in the static tensile test. Figure 4 shows an example of the stress-elongation characteristics for samples with a thickness of 1 mm, for which the tension direction was, accordingly, perpendicular (Figure 4a) and parallel (Figure 4b) to the rolling direction.

Based on the presented material characteristics of EN AW-2024 T351 aluminum alloy, it was found that the form of the tensile curves was typical for this group of aluminum alloys. The resulting elongation was *ε* = 15.64% for the perpendicular direction and *ε* = 15.69% for the parallel direction with the ultimate tensile strength *UTS R_m_* = 432.31 MPa (perpendicular direction) and *R_m_* = 437.12 MPa (parallel direction).

In order to determine the influence of rolling and the ratio of the tension direction to the rolling direction on the mechanical properties of the EN AW-2024 T351 aluminum alloy, the results obtained during the static tensile test were analyzed.

**Figure 5:**
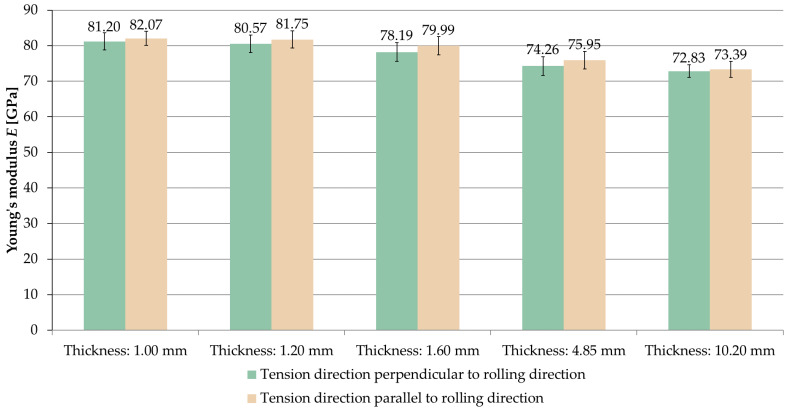
Young’s modulus *E* depending on the thickness of the sample and the ratio of the tension direction to the rolling direction.

Figure 5 shows the average values of Young’s modulus *E* received for the analyzed sample thicknesses and the relationships between the tension direction and the rolling direction. On the basis of the results, it was found that for all thicknesses, the value of the longitudinal modulus of elasticity *E* was greater in the range of 0.7–2.3% for the tension parallel to the rolling direction than for tension in the perpendicular direction.

**Figure 6:**
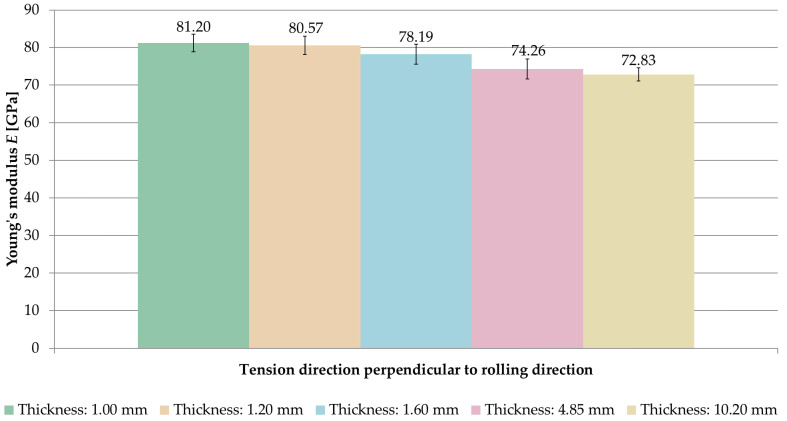
Comparison of Young’s modulus *E* for samples stretched perpendicular to the rolling direction.

**Figure 7:**
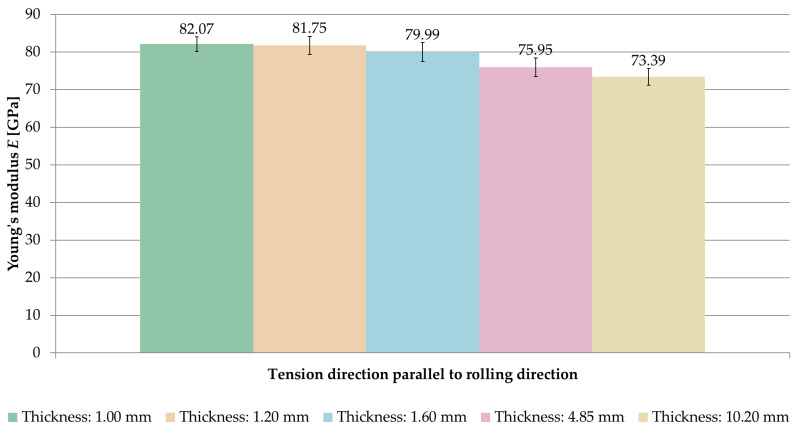
Comparison of Young’s modulus *E* for samples stretched parallel to the rolling direction.

Figure 6 and Figure 7 present a comparison of the Young’s modulus *E* for samples stretched in perpendicular and parallel directions to the rolling direction, respectively.

When analyzing the obtained results, it was observed that the longitudinal modulus of elasticity *E* increased with decreasing sample thickness.

For samples where the tension direction was perpendicular to the rolling direction, the highest value of Young’s modulus was *E* = 81.20 GPa (thickness of 1 mm), while the smallest one was *E* = 72.83 GPa (thickness of 10.2 mm). In the case of samples stretched parallel to the rolling direction, an analogous regularity was observed. The highest value of the longitudinal modulus of elasticity *E* was recorded at a thickness of 1 mm (*E* = 82.07 GPa) and the smallest one at 10.2 mm (*E* = 73.39 GPa). In both cases, greater by about 10% of the Young’s modulus, *E* was obtained with a thickness of 1 mm compared to a thickness of 10.2 mm.

**Figure 8:**
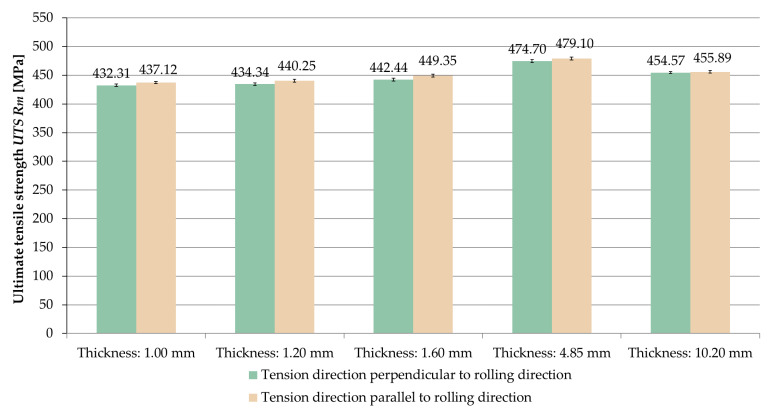
Ultimate tensile strength *UTS R_m_* depending on the thickness of the sample and the ratio of the tension direction to the rolling direction.

The averaged values of ultimate tensile strength *UTS R_m_* obtained for the tested sample thicknesses and the relationships between the tension direction and the rolling direction are shown in Figure 8.

Analyzing the results, it was noticed that for the samples stretched parallel to the rolling direction, the ultimate tensile strength *UTS R_m_* was greater in the range of 0.2–1.6% than for samples stretched perpendicular to the rolling direction.

**Figure 9:**
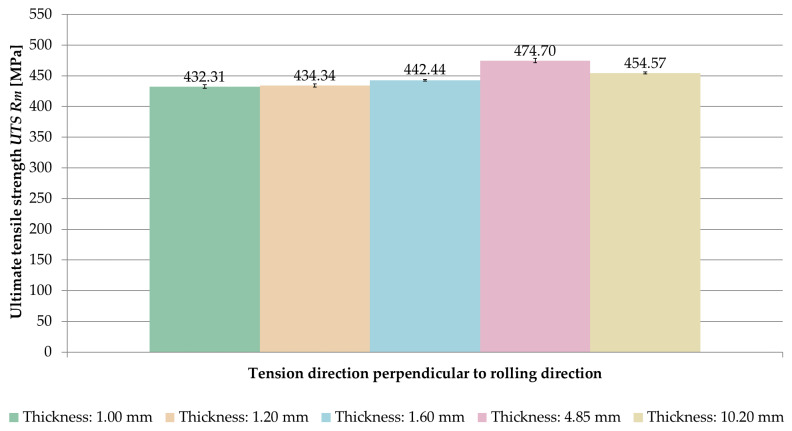
Comparison of ultimate tensile strength *UTS R_m_* for samples stretched perpendicular to the rolling direction.

**Figure 10:**
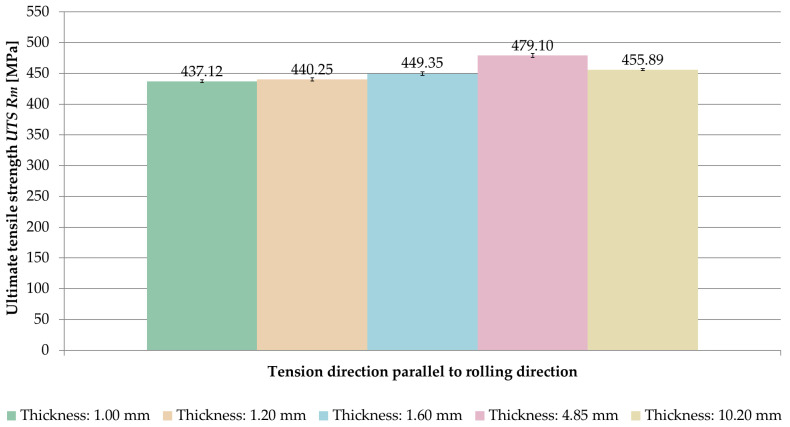
Comparison of ultimate tensile strength *UTS R_m_* for samples stretched parallel to the rolling direction.

The comparison of averaged values of ultimate tensile strength *UTS R_m_* for samples stretched in perpendicular and parallel direction to the rolling direction is shown in Figure 9 and Figure 10.

On the basis of the obtained results, it was found that the ultimate tensile strength *UTS R_m_* increased with increasing sample thickness, but only up to a certain point. The maximum value was recorded at a thickness of 4.85 mm, for which the ultimate tensile strength *UTS* was *R_m_* = 474.70 MPa (perpendicular direction) and *R_m_* = 479.10 MPa (parallel direction).

**Figure 11:**
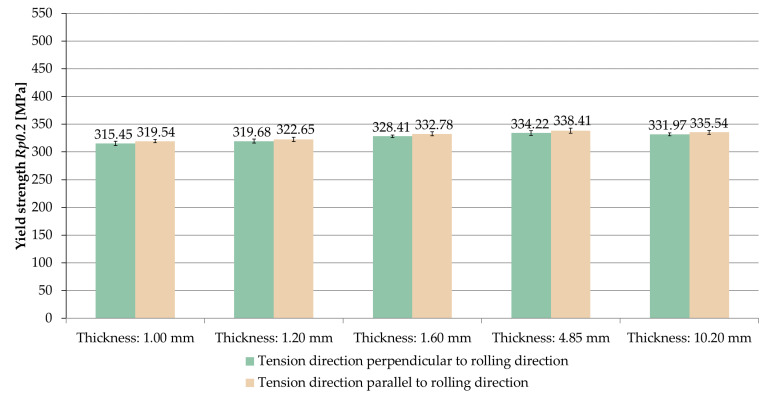
Yield strength *R_p_*_0.2_ depending on the thickness of the sample and the ratio of the tension direction to the rolling direction.

The averaged values of yield strength *R_p_*_0.2_ obtained for the tested sample thicknesses and the relationships between the tension direction and the rolling direction are shown in Figure 11.

The same relationship was found as in the case of ultimate tensile strength *UTS R_m_*. On the basis of the results, it was noticed that for the samples stretched parallel to the rolling direction, the yield strength *R_p_*_0.2_ was greater in the range of 0.9–1.3% than for samples stretched perpendicular to the rolling direction.

**Figure 12:**
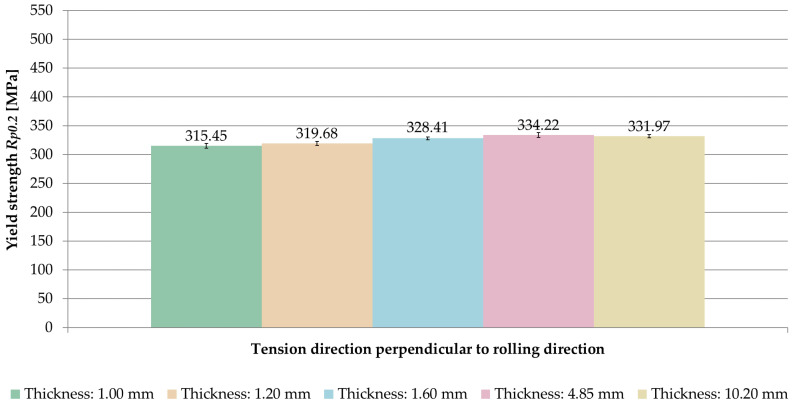
Comparison of yield strength *R_p_*_0.2_ for samples stretched perpendicular to the rolling direction.

**Figure 13:**
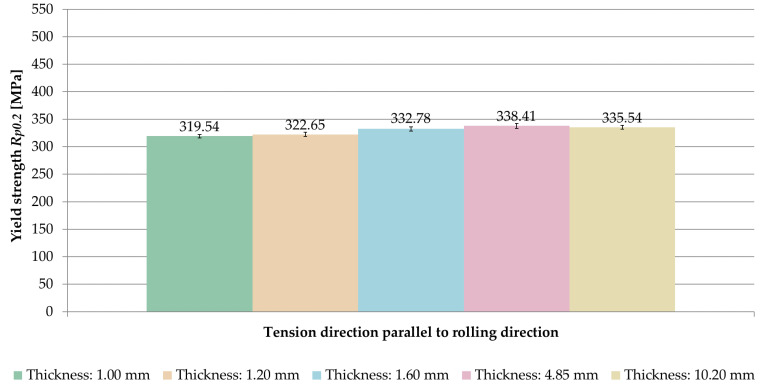
Comparison of yield strength *R_p_*_0.2_ for samples stretched parallel to the rolling direction.

A comparison of the averaged values of yield strength *R_p_*_0.2_ for samples stretched in perpendicular and parallel direction to the rolling direction is shown in Figure 12 and Figure 13. Based on the results, it was found that the yield strength *R_p_*_0.2_ increased with growing sample thickness, but, also, only up to a certain point. The maximum value was recorded at a thickness of 4.85 mm, for which the yield strength was *R_p_*_0.2_ = 334.22 MPa (perpendicular direction) and *R_m_* = 338.41 MPa (parallel direction).

In the study, the complete, randomized, statistical program was used to assess the influence of the examined independent variables. The null hypothesis *H*_0_ about the lack of influence of the input factor on the result factor was adopted. Statistic *F* Fisher–Snedecor was used and test value *F* was determined from the dependence (2):

(2) $$F=\frac{\sum_{i=1}^{p}n_{i}{\left({\bar{y}}_{i}-\bar{y}\right)}^{2}\left(n-p\right)}{(\sum_{i=1}^{p}\sum_{j=1}^{q}{\left(y_{ij}-\bar{y}\right)}^{2}-\sum_{i=1}^{p}n_{i}{\left({\bar{y}}_{i}-\bar{y}\right)}^{2})\left(p-1\right)},$$

where *n_i_* is the number of measurements of the input factor at a given level, *n* is the total number of measurements, $\bar{y}$*_I_* is the mean of the measurement results in *i*-line, $\bar{y}$ is the mean of the results from all measurements, *y_ij_* is the value of *j*, which is a factor of the result on the level *i*, and *p is the* number of levels of variation of the input factor.

The value of the statistic *F* compared with the critical value $F_{kr\left(\alpha ,f_{1},f_{2}\right)}$ determined on the basis of the adopted level of significance *α* and the number of degrees of freedom *f*_1_ and *f*_2_ is designated, respectively, for the numerator (3) and the denominator (4):

(3) $$f_{1}=f_{n}=p-1,$$

(4) $$f_{2}=f_{d}=n-p.$$

If the calculated value of the test statistic *F* was greater or equal to the critical value $F_{kr\left(\alpha ,f_{1},f_{2}\right)}$, the null hypothesis *H*_0_ was rejected and the influence of the examined factor was considered as significant at the assumed significance level *α*.

Table 7 and Table 8 present the results of the analysis of the significance of the influence of the tested variables, i.e., the sample thickness and the ratio of the tensile direction to the rolling direction on Young’s modulus *E* and ultimate tensile strength *UTS R_m_*.

Based on the results of the verification of the significance of the influence of the analyzed independent variables at the adopted significance level of α = 0.05, we found:

- significant influence of the sample thickness and no influence of the relationships between the tension direction and the rolling direction to Young’s modulus E, and
- significant influence of both the sample thickness and the relationships between the tension direction and the rolling direction on the ultimate tensile strength UTS Rm.

### 3.2. Deformation Research

**Figure 14:**
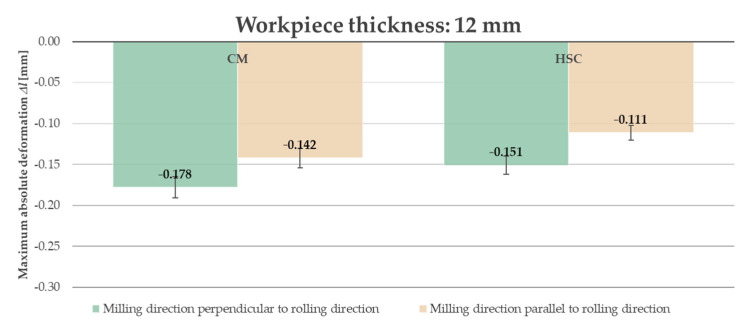
Comparison of the maximum absolute deformations Δ*l* depending on the ratio of the milling direction to the rolling direction for the semi-finished product with a thickness of 12 mm.

**Figure 15:**
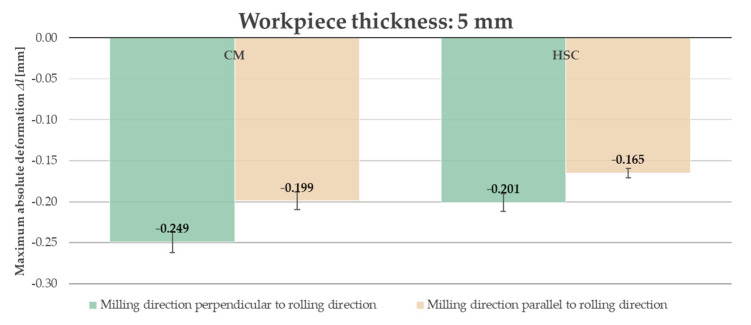
Comparison of the maximum absolute deformations Δ*l* depending on the ratio of the milling direction to the rolling direction for the semi-finished product with a thickness of 5 mm.

**Figure 16:**
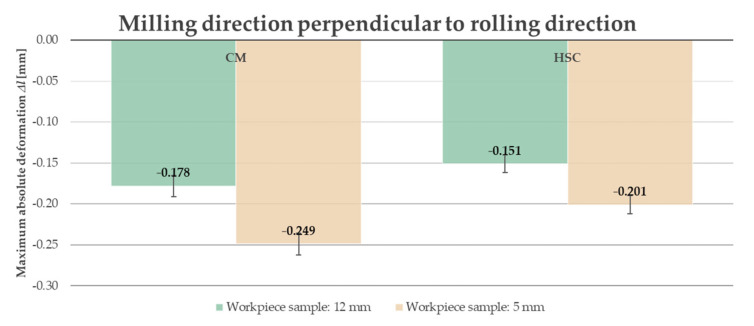
Comparison of the maximum absolute deformations Δ*l* depending on the semi-finished product thickness for milling in perpendicular direction to rolling direction.

**Figure 17:**
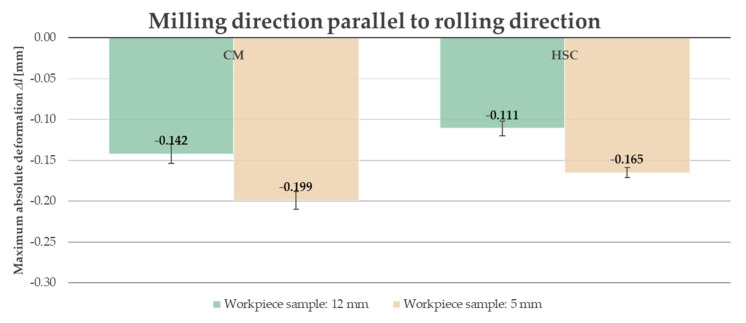
Comparison of the maximum absolute deformations Δ*l* depending on the semi-finished product thickness for milling in parallel direction to rolling direction.

Due to the fact that, in industrial practice, thin-walled elements undergo significant deformation at the moment when the clamping forces are removed, it was decided to present the results after unfastening the workpiece from the clamping device. Furthermore, analyzing the obtained results, i.e., the course of the absolute deformation Δ*l* as a function of the sample length *l*, it was observed that its maximum value occurred in the center of the sample (around *l* = 82.5 mm) in all cases. A characteristic “negative” deflection was noted, denoting the location of the deviation below the adopted “zero” line, meaning lack of deformation. Hence, the results of the deformation tests were developed and presented in the form of bar graphs referring to the recorded maximum values. The abbreviation “CM” determines conventional machining and HSC denotes High-Speed Cutting. Figure 14 and Figure 15 present a comparison of the maximum absolute deformations Δ*l* depending on the ratio of the milling direction to the rolling direction for the tested semi-finished product thicknesses, while Figure 16 and Figure 17 show the maximum absolute deformations Δ*l* as a function of the semi-finished product thickness for milling in the perpendicular and parallel directions to the rolling direction, respectively.

On the basis of the obtained results, it was found that the absolute deformations Δ*l* were higher by about 20–30% for samples that were milled in the perpendicular direction to the rolling direction in relation to the samples milled in parallel direction. Moreover, it was observed that the greater values of the absolute deformations Δ*l* were obtained by approximately 35–45% for samples made of a semi-finished product with a thickness of 5 mm. It was also found that, in order to minimize the deformation, High-Speed Cutting should be used because the absolute deformations Δ*l* were smaller, in the range from 15% to 30%, depending on the analyzed variant (in comparison to conventional machining).

A similar verification of the significance of the influence was also performed in the case of absolute deformations Δ*l*. Based on the obtained results, at the adopted significance level of α = 0.05, we found significant influence of the ratio of the milling direction to the rolling direction, semi-finished product thickness, and finishing machining on the absolute deformations Δ*l*.

## 4. Discussion

The analysis of the results obtained in the tensile tests allowed for the conclusion that the thickness of the element and the ratio of the tension direction to the rolling direction have impact on the mechanical properties of the EN AW-2024 aluminum alloy in the T351 condition. It is related to the influence of the rolling process on the surface layer. For samples with the smallest thickness, the influence of the hardened, textured surface layer was significant, which translated into higher values of Young’s modulus *E* compared to samples of greater thickness. A similar relationship was found in the case of ultimate tensile strength *UTS R_m_* and yield strength *R_p_*_0.2_, but it was also noted that these parameters increased with increasing sample thickness only up to a certain point. The maximum values were obtained at 4.85 mm.

On the basis of the obtained results, it can also be concluded that the post-machining stresses influenced the deformation values of the thin-walled elements. The type of these stresses (tensile or compressive stresses) depends on the nature of the dominant interactions during machining. The model of thermal effects’ characteristic for abrasive machining may also dominate in machining at very high cutting speeds, e.g., during HSC, while the mechanical model was typical for machining with tools with a defined blade geometry. Thermal actions generated positive tensile stresses in the surface layer, while mechanical actions generated negative compressive stresses. In practice, the stress state in the surface layer is the result of the interaction of these two models, but their intensity may be different. It was evident in the case under consideration. For conventional machining, for which the mechanical model seemed to be the dominant, the generated compressive stresses accumulated with tensile residual stresses in the lower part of the semi-finished product (bottom wall opposite to the machined wall), increasing the deformation values. In the instance of High-Speed Cutting, the effect of the thermal model became more important. The compressive stresses in the machined surface layer decreased, which was manifested by a reduction in the deformation of the thin-walled bottom.

An important aspect is also the difference between the post-machining deformations obtained for samples milled in perpendicular and parallel directions to the rolling direction, which can be related to the results of tensile tests. For samples that were stretched parallel to the rolling direction, higher values of Young’s modulus *E*, yield strength *R_p_*_0.2_ and ultimate tensile strength *UTS R_m_* were noted (the influence on ultimate tensile strength *UTS R_m_* was statistically confirmed), while in the deformation test, the samples milled perpendicular to the rolling direction were more deformed. It could mean that elements with higher yield strength *R_p_*_0.2_ and ultimate tensile strength *UTS R_m_* underwent less deformation. Therefore, the so-called “technological history effect” of the semi-finished product is very significant. Moreover, greater deformations were noted for samples made of semi-finished product with a thickness of 5 mm. In this case, it was probably the result of the influence of the surface layer, which at lower thicknesses had a higher percentage of contents than in thicker samples. On the basis of the obtained results, it can be concluded that the value of residual stresses also depended on the thickness of the rolled materials. Additionally, together with other features of the surface layer, they changed the mechanical properties of the rolled materials and anisotropy, with these properties depending on the rolling direction. Therefore, the mechanical properties had an influence on the resulting post-machining deformations.

Referring to the earlier publications of the authors, the research focused on minimization of the deformation of thin-walled elements by means of a properly conducted milling process. Due to the interest in this subject area, both on the side of scientists and industry, the tensile tests were conducted, aimed at explaining the cause-and-effect relationship between the mechanical properties of the material and the occurring post-machining deformations. In previous publications (e.g., [41,43,44]), the authors tested samples made of semi-finished product in a form of rolled plate with a thickness of 10 mm. In this study, the results for thicknesses of 5 mm and 12 mm were verified, respectively. It was also to confirm whether the results obtained for thickness of 10 mm can be extrapolated to a different geometry. Based on the results, similar relationships were found. Additionally, it was noted that samples made of a semi-finished product with a lower thickness underwent greater deformations than those made of a thicker rolled plate. It was probably related to the influence of the surface layer, which, for a semi-finished product with lower thickness, had a higher percentage of content. Moreover, this paper tried also to explain why samples milled parallel to the rolling direction deformed less in comparison to samples milled in a perpendicular direction. It is related to the anisotropy of the mechanical properties. For samples stretched parallel to the rolling direction, greater values of Young’s modulus *E*, ultimate tensile strength *UTS R_m_*, and yield strength *R_p_*_0.2_ were found than for samples stretched perpendicularly. The anisotropy of the structure was presented in [44] and the possibility of minimization of post-machining deformations was shown with appropriately selected machining techniques (combination of conventional machining and HSC) in conjunction with milling in the parallel direction to the rolling direction [19]. The publication [43] presented the reduction of deformations, thanks to the application of pre-machining consisting of the removal of the textured surface layer. The present test results explain some cause-effect relationships and indicate a further possibility of deformation minimization with the appropriately selected thickness of the semi-finished product, in this case, rolled plate. In another paper [41], it was found that seasoning does not reduce post-machining deformations, while, in [38], an analysis of deformations in the aspect of the cutting tool used was performed, on the basis of which it was observed that it is possible to reduce the deformation with an appropriately selected cutting tool. Summarizing, in this paper an attempt was made to explain the causes of the deformations by referring to the mechanical properties of the material used.

## 5. Conclusions

The conducted research and the analysis of the obtained results allowed for the formulation of the following conclusions:

- The thickness of the rolled, semi-finished products had a significant influence on the strength properties of the tested material. With the increase in the thickness of the samples, the value of Young’s modulus *E* decreased with a simultaneous increase in yield strength *R_p_*_0.2_ and ultimate tensile strength *UTS R_m_*, but these parameters increased only to a certain point. It was the effect of the textured surface layer, which had a greater impact on elements of less thickness.
- The mechanical properties of the semi-finished product were different for the analyzed directions in relation to the rolling direction. It is related to the well-known phenomenon of anisotropy of rolled materials.
- Greater deformations were noted for samples made of the semi-finished product with thickness of 5 mm in comparison to a thickness of 12 mm. It was probably the result of the influence of the surface layer, which, at lower thicknesses, had a higher percentage of contents than in thicker samples.
- The nature and value of deformation of the machined element, in addition to the phenomena of anisotropy and residual stresses resulting from the “technological history” of the semi-finished product, depend also on post-machining stresses.
- Depending on the adopted technological parameters, one of the models, i.e., thermal or mechanical, may be the dominant one during machining. These models are characterized by a different character of post-machining stresses. The thermal model generates tensile stresses, while the mechanical generates compressive stresses in the surface layer.
- The use of higher cutting speeds (High-Speed Cutting) resulted in a reduction of post-machining deformations.
- The possibility of influencing the deformation values by changing the technological parameters in combination with the knowledge of the residual stresses after rolling has a very significant utilitarian significance. By designing the technological process in an appropriate way, it is possible to reduce the deformation of the machined elements.
- The presented technological guidelines can also be a source of knowledge for aviation companies, which, thanks to their application, influence minimizing post-machining deformation of thin-walled elements.

## Figures and Tables

**Table 1:** Chemical composition and selected properties of EN AW-2024 T351 aluminum alloy [46,47].

Numerical Indication					EN AW-2024					
Chemical Indication					EN AW-AlCu4Mg1					
Chemical composition [%]										
Si	Fe	Mg	Cu	Mn	Zn	Cr	Zr + Ti	Ti	Other	Al
≤0.5	≤0.5	1.2–1.8	3.8–4.9	0.3–0.9	≤0.25	≤0.1	≤0.2	≤0.15	≤0.15	Rest
**Selected properties**										
Density *ρ* [g/cm^3^]		Young’s modulus *E* [GPa]		Ultimate tensile strength *UTS R_m_* [MPa]		Yield strength *R_p_*_0.2_ [MPa]		Brinell hardness		
2.78 g/cm^3^		73 GPa		469 MPa		324 MPa		120 HB		

**Table 2:** Calculation of the sample size *n* in tensile tests.

	Young’s Modulus *E* [GPa]	Ultimate Tensile Strength *UTS R_m_* [MPa]
** $\mathrm{Mean}\;\bar{x}$ **	72.125	431.375
**Standard deviation *σ***	4.72621	19.25954
** $\mathrm{Variance}\;S_{x}^{2}$ **	22.33706	370.92988
**Assessment accuracy *A***	3.60625	21.56875
**Factor *t* (*α* = 0.05; *f* = 7)**	2.365	
**Sample size *n***	**9.61**	**4.46**

**Table 3:** Technical specifications of the tools used [51,52].

Tool	Kennametal	Sandvik
**Symbol**	25A03R044B25SED14	R216.33-16040-AC32U
**Cutting insert symbol**	EDCT140416PDFRLDJ	-
**Material of cutting inserts/blades**	KC410M	H10F
**Number of cutting edge *z***	3	3
**Working part diameter *d* [mm]**	25	16
**Total length *L* [mm]**	101	92
**Maximum depth of cut *a_pmax_* [mm]**	14.6	32
**Grip part diameter *D* [mm]**	25	16

**Table 4:** Applied technological parameters during finishing.

Technological Parameters	Conventional Machining	High-Speed Cutting
**Depth of cut *a_p_* [mm]**	0.4	0.4
**Milling width *a_e_* [mm]**	12	12
**Cutting speed *v_c_* [m/min]**	200	1200
**Feed per tooth *f_z_* [mm/tooth]**	0.02	0.02
**Rotational speed *n* [rpm]**	3979	23,873

**Table 5:** Technical specification of the Sylvac CL44 sensor [54].

Parameter	Value
Plunger length [mm]	36.5
Measurement range [mm]	±0–0.5
Resolution [mm]	0.001
Accuracy [mm]	0.01
Pressure [N]	0.07 (±15%)

**Table 6:** Calculation of the sample size *n* in tensile tests.

	Absolute Deformation Δ*l*, mm	
	Tool Feed Direction Perpendicular to Rolling Direction	Tool Feed Direction Parallel to Rolling Direction
** $\mathrm{Mean}\;\bar{x}$ **	−0.2465	−0.207
**Standard deviation *σ***	0.01319	0.00910
** $\mathrm{Variance}\;S_{x}^{2}$ **	0.00017	0.00008
**Assessment accuracy *A***	−0.01675	−0.01230
**Factor *t* (*α* = 0.05; *f* = 4)**	2.776	
**Sample size *n***	**4.67**	**4.07**

**Table 7:** The results of the analysis of the significance of the influence of the studied variables on Young’s modulus *E*.

		*F*	*F_kr_*	Result	Conclusion
Verification of the significance of the influence of sample thickness (*f*_1_ = 4, *f*_2_ = 45)					
**Ratio of tension direction to rolling direction**	**Perpendicular**	24.47	2.58	***F > F_kr_***	**Significant influence of sample thickness**
	**Parallel**	26.88			
Verification of the significance of the influence of rolling direction (*f*_1_ = 1, *f*_2_ = 18)					
**Sample thickness**	**1 mm**	0.79	4.41	***F < F_kr_***	**Lack of influence of rolling direction**
	**1.2 mm**	1.17			
	**1.6 mm**	2.36			
	**4.85 mm**	2.18			
	**10.2 mm**	0.43			

**Table 8:** The results of the analysis of the significance of the influence of the studied variables on ultimate tensile strength *UTS R_m_*.

		*F*	*F_kr_*	Result	Conclusion
Verification of the significance of the influence of sample thickness (*f*_1_ = 4, *f*_2_ = 45)					
**Ratio of tension direction to rolling direction**	**Perpendicular**	479.69	2.58	***F > F_kr_***	**Significant influence of sample thickness**
	**Parallel**	412.28			
Verification of the significance of the influence of rolling direction (*f*_1_ = 1, *f*_2_ = 18)					
**Sample thickness**	**1 mm**	17.46	4.41	***F > F_kr_***	**Significant influence of rolling direction**
	**1.2 mm**	25.95			
	**1.6 mm**	48.16			
	**4.85 mm**	7.94			
	**10.2 mm**	4.99			

## Data Availability

The data presented in this study are available on request from the corresponding author.
